# Illinois State Dental Society

**Published:** 1872-07

**Authors:** 


### PROCEEDINGS OF SOCIETIES.

### Illinois State Dental Society.

The eighth annual meeting of this society was held at Chicago, May 14th, 1872. Dr. O. Wilson, of Aurora, presided, and over seventy-five of the active members were present.

The President, Dr. Wilson, delivered the annual address, which was listened to with marked attention, and was full of very interesting statistics.

The first subject for discussion was opened by the reading of an essay by Dr. E. D. Swain, of Chicago, on Mechanical Dentistry.

He showed the necessity of a knowledge of dentistry, physiology and anatomy, to the mechanical dentist, and deplored the fact that this branch of the profession is being discarded by the leading men, and with it the only truly surgical part of dentistry is also cut off. The same hands that restore defects in the natural organs should give their attention to at least the arrangement and selection of the artificial ones, as they alone can imitate nature, knowing the requirements of the case before them; the purely mechanical execution may be left to other hands. There is more opportunity for the exercise of artistic skill in the use of single gum and plain teeth than in the use of blocks. Among the legitimate means at the disposal of the artist for the imitation of nature were enumerated a certain degree of irregularity, such as overlapping laterals, etc., gold fillings, worn crowns, necks shaved to represent wasted alveolus, etc. Generally recommends zinc dies, sometimes uses a Babbit metal model to finish upon. Clasps have been a failure mainly on account of their improper adjustment. It is essential to get a perfect impression of the tooth to be clasped; this can be obtained by fitting a clasp of sheet lead or tin over the tooth, splitting this on two sides, and filling with plaster of Paris; if necessary in removing, the plaster can be broken with the slit in the cap, the cap bent apart, and after removal brought together again. After the clasp and plate are fitted their relative position can best be secured by placing them in their position in the mouth, and with a spatula laying plaster upon the joint, and allowing this to set before removal; does not prefer clasps, but believes there are cases where nothing else will answer.

*Dr. G. O. Howard*, of Galena, asked if Dr. Swain had ever made clasps by winding platina wire around a plaster tooth and flowing gold on this.

*Dr. Swain* replied, he had not, but thought it an admirable way.

*Dr. Sherwood*, of Chicago, believes the manufacture of teeth in blocks a great advance toward perfection; atmosperic pressure plates can be made in all cases—clasps are never admissible.

*Dr. Kilbourne*, of Aurora, believes clasps admissible; has worn a clasp himself since '52 without injury.

*Dr. Honsinger*, of Chicago—Clasps may be safely used; they are generally made too soft; makes his of gold alloyed with the twelfth of platinum.

*Dr. Sturgiss*, of Quincy, uses that which is best in the case before him; clasps will do some harm, but so will rolling suction plates.

*Dr. Swain* has had some hopes of aluminum, but has had some plates returned to him with holes eaten through them· has melted and refined the plates bought at the depots, and rolled out his own plate, and obviated the difficulty. Has made plates of this substance combined with Ingersoll's Am. Platina; this makes nice work and is free from galvanic action.

*Dr. Kilbourne* said his experience with aluminum was like Dr. Swain's, holes eaten through plates; hopes this may be overcome. Continuous gum work is the best, and next to it gold with rubber attachments.

*Dr. C. B. Rising*, of Rockford—Rubber ought not to be condemned because the idyosyncrasies of some persons may be opposed to it. Where there is one person who can not use rubber there are five hundred who can. Thought celluloid bids fair to supercede it. Has never had patients complain of taste of camphor in these plates.

*President Wilson's* experience with aluminum was the same as Dr. Swain's—plates returned full of little holes.

*Dr. Dean*, of Pekin, stated his experience with aluminum the same—has abandaned its use. (Dr. Dean's plates were cast, and Dr. Swains', Kilbourne's and Wilson's swedged.)

The whole forenoon of the next day was set apart for clinical operations, which were held at S. S. White's dental depot.

In the afternoon Dr. G. O. Howard, of Galena, read an essay on "Dental Quacks." The paper defined the term, and illustrated several varieties of the species, and finally said that even college graduates are not entirely free from being amenable to charges of quackery, and quoted several lengthy newspaper advertisements as a basis for this assertion. As a remedy he recommended the education of the public upon this subject, and commended the publication of the *Dental Mirror* by the St. Louis Dental Society, as a good move in that direction.

*Dr. Kitchen*, of Galva, said that men sometimes style themselves D. D. S. in advertisements, who really are not graduates of dental colleges.

*Dr. M. S. Dean*, of Chicago, then read an essay on "Sound Practice Sound Philosophy," of which the following are brief extracts:

"Our practice to be successful must be based on sound philosophy, that is upon the knowledge of those principles and laws that govern the materials or agents with which we operate, and also those upon which we operate.

* * * In the filling of very fragile teeth where there is danger to be apprehended, as checking the enamel or splitting the bicuspids, it is more philosophical to use the heavy leaden mallet than light steel or wooden ones, as the latter have greater *vis viva* in proportion to their momentum. When the tooth is very brittle cueniform pluggers should not be used as the tapering points act as wedges, and may either check the enamel or split the tooth. The same result might be effected by driving the gold too forcibly into cavities that diminish from their orifices. This evil may result from the use of the heavy lead mallet, but the lighter mallet would be more capable of producing this result. It is not sound practice to draw the gold over the margins of a cavity with mallet or burnisher. * * * In filling a large crown cavity with surrounding walls unbroken, the central portion should be kept higher than the peripheral, especially as the work approaches completicn. * * *

Gold should not be forced uuwillingly into any portion of a filling where cohesion is important, nor against the walls of a cavity.

The pellet and plug must unite by "first intention," and before the gold has been rolled from place to place and thus become partially condensed or knotted. Large pellets should be packed with correspondingly large points, and afterwards *condensed* with sharper ones. * * * It is sufficient if the walls of the cavity are absolutely protected, and the gold sufficiently consolidated to resist the force of mastication.

It is more philosophical not to compact the fillings of very *large cavities* perfectly. In *some respects* a porous filling is better than a perfectly dense one. All gold fillings are porous to some extent, and there is little danger of getting them too compact. * * * It can not be possible to pack gold more accurately against *concave* walls with instruments having a smooth, flat surface, than with a serrated convex one. If the surface of the filling could be kept flat no points could be better adapted to the purpose of making dense fillings; but this can rarely be done, and where it is not, no points are better adapted to shingle or bridge over the interstices and deceive the operator. It has recently been asserted by a writer who uses the "steel mallet and smooth points," that with these, operations are performed, giving no pain to the patient, and with no risk of fracturing the enamel; and that the fillings are harder and better adapted to the margins of the cavity. Whether this magic power exists mostly in the steel mallet or the smooth points, or whether they are equally efficacious in producing these *desirable* results, and in averting the contrary ones, the writer has neglected to state. Most dentists place a layer, or cushion as it were, of gold between the instrument and the enamel. If this is done these points would as readily fracture the enamel as if they were filney serrated. If this inference is correct we are forced to conclude that this charm belongs to the peculiar properties of the steel of which this wonderful mallet is made, and we are warranted in this conclusion by the assurance of the writer that it is *non-vibrating.* * * * By beating the gold under a smooth point plugger, it is drawn snugly and perfectly against the walls and margins of the cavity; on the same principle as a cubic slug is made to adjust itself to the sides of a gun-barrel, when vigorously rammed down with a steel ramrod. If the enamel were made of steel, this would be sound practice, provided our patients were made of steel also. * * *

Successin the treatment of alveolar abscess depends, in a great measure, upon sound philosophy. * * * One or two thorough applications of creosote or carbolic acid are usually sufficient. The continued and daily application afterwards often produce the troublee instead of relieving it. * * * Those having a fistulous opening are easily treated and quickly cured, as the irritating particles may be washed out by injections. Hence great care should be exercised when there is no opening, to remove patiently and thoroughly all extraneous matter from the canals of roots, before therapeutic remedies are applied. * * * The universal practice of restoring the contour of the teeth is not sound practice, in many cases it is important to masticulation and the articulation of the voice, and also to the beauty of these organs; but the practice was becoming too universal. Many cases were instanced where they must fail, and where fillings having an inclining surface, so that the force of mastication would be in a direction to retain them, would be more philosophical.

*Prof. Judd*, of St. Louis, could find but little with which to disagree. He has used the common wood, the light and heavy lead, the steel and the soft rubber mallet, but now uses the steel mallet exclusively. Experience rather than philosophy should guide us. Philosophers are not of much account when they do not accord with experience. The mallet that gives the leat s*vis viva* with the least momentum will give the least pain.

*Dr. Crouse*—Can not understand how the steel mallet can be preferred in St. Louis when his own patients prefer the leaden. Was glad the essayist called attention to danger of cracking enamel. This is increased by a quick, sharp blow of a light mallet. Calls attention to trimming down the gold even with the edges. Soft foil adapts itself better to the walls and edges than cohesive; and he always likes to use a layer of soft foil next to the walls and edges. Favors serrations even deeper than Varney's. There is no good derived from frequent repititions of treatment in diseased teeth; thoroughness the first time is the greatest essential.

*Prof. Morrison*, of St. Louis, was among the first to adopt the steel mallet; had used lead before; desired to know the difference in weight between the steel and leaden mallets, as used by Dr. Crouse.

*Dr. Crouse*—Should think them of about the same weight.

*Dr. G. V. Black*, of Jacksonville, thinks there is as much in the assistant's hand becoming accustomed to a particular mallet, as in the difference between mallets; dislikes to hear teeth spoken of as dead in which only the pulp is dead.

*Dr. DeCrow*, of Quincy, believed with Dr. Block, that changing from one mallet to another objectionable, the one longest used by the assistant will give least pain.

*Dr. Sherwood*—Used lead mallet up to the great fire—immediately after used a three-ounce tack hammer with good success.

*Dr. Wilson*, of Bloomington, thinks the greatest importance lies in fixing the first pellet, and the next in cohesion, at the first touch.

*Dr. W. W. Allport*, of Chicago, commends the thoroughness of the essay; is especially pleased to see the position taken upon the manner of keeping the gold while compacting—keeping the center higher than the edges. Dr. Dwinelle and others taught, when crystal gold first came into use, that the margins must be kept higher. He believed this to be the error, and spoke of it first some ten years ago, and again at the meeting of the American Dental Association, at Cincinnati, and also before this Society, at its last meeting, in this city. This is the first time he has ever heard this manner advocated by any one else publicly. Said Dr. Black had said that because merely the pulp was dead the tooth was not dead also, but he would go farther and state that the dentine may be dead to all intents and purposes, and yet the pulp be alive.

*Dr. Kennicott*, said he believes that he is the original advocate, as he is the oldest practitioner of the present method of filling teeth with smooth pointed pluggers and adhesive foil. The too wise men of the nation, who are now claiming to be the originators of this method, not only got their first ideas from him, but he also furnished them with their first instruments. He had explained and illustrated his method at the session of the American Dental Association, at Cincinnati, and also at the meeting of the Society held in this city in 1867, and now thinks it rather rough to find others stealing his thunder, and making capital out of his ideas.

He had also used instruments that were neither smooth nor serrated points, and which in his peculiar method, for which he claims originality and marked success, must be hard and highly polished, not merely worn smooth and constructed with undulations or slight elevations upon the impinging surface.

These being used with a rotary or oscillating motion, break down and condense the gold, making a more uniformly, consolidated and durable filling than can be obtained with serrated points.

*Dr. Kilbourne* uses a broken pointed plugger more than any other; considers its granular surface better than any artificial serration.

After the transaction of some other business, the Society adjourned until 9 o'clock next day.

*Dr. S. L. Edwards* read an essay on "Our Failures," in this he drew a very vivid picture of a practitioner who is so overrun with work that his system is like the miller who turns out his grist with particular regard for the toll, but who still must attend to all calls in pe son because "there is no competent neighboring practitioner. This man sends gold plates to the jeweler, and silver plates to the tin-smith, if left with him for repairs. The essayist here inserted an account of the operations performed by this man in one day. Next day a flaming notice announces that Dr. Goodlar extracted 93 teeth yesterday, although it was not a good day for toothache. This he said was but a faint recollection from recent observation. All impositions practiced upon the public, by any one within the profession may fairly be imputed to the most worthy, an one man ought to screen himself by retorting, "Am I my brother's keeper?"

To check the further progress of ignorance in the profession, he proposed that the fundamental law of this society be so amended as to prescribe the least space of time allowed any member for which to take a student. Dentists should be free from all moral and physical stain, so that every word, act or thought may prove a constant, pure and illuminating power. They should place themselves on record with those noble men of the medical profession who deny alcohol a place in materia medica, so that patients may realize what they have a right to expect, a clear eye, a steady hand, and an unclouded brain.

*Dr. Crouse*—This case shows the necessity of selecting better material for our students. We take students merely for selfish purposes, and by so doing make an alms house of the profession. We ought to encourage young men of capacity to enter it, but such young men must not expect to pay their way while pursuing their studies; they should have at least an academic education, and a certain amount of native talent.

*Dr. Sturgiss* did not believe in shutting the door to any who may wish to enter. If the power to become a shining light dwells within a man, do not attempt to choke him off.

*Dr. Crouse* said his idea was, that we ought not to take the poor, because they would act as menials for us, but rather if we find that he has the right material in him, we should assist him by educating him gratuitously.

*Dr. Kitchen*, of Galva—No matter how well qualified a man may be, if he lacks in morality and integrity, he will disgrace his profession.

*Dr. Kennicott* called the attention of the society to the fact that in Canada, the practice of dentistry is regulated by law; and not only has the practitioner to appear before a Board of Examiners appointed and paid by the Government, before he is allowed to practice, but a candidate for studentship has also to appear before this Board before he is permitted to be indentured. Thinks this is a wholesome example to us, and we ought to profit by it.

*President Wilson*—No obstacles and trammels should be thrown in the pathway of scientific research or any laudable enterprise; yet, safeguards that shall certainly secure excellent qualifications, though they may prove a stumbling block to some, are invaluable. Had an application from an M. D., who "counts" that he might find time to extract and fill, as in his locality there is no dentist, if he knew well how. Advised him to practice only one profession, suggesting the time necessary for thorough drill.

*Dr. K. B. Davis*, of Petersburg, then made an essay on "Dental Societies," of which the following are a few thoughts:

While he does not detract from the just claims of the dental colleges and journals as elevators of dental science, yet he claimed that the free interchange of mind with mind at the meetings of dental societies, should be given the honor of exerting a more marked and direct influence. The clinics at these meetings have lifted the mist and fog of perplexity from many whose pupilage and after observation was limited; and these reaching a greater number, do more good than the colleges. Those who do not attend the meetings because they can not afford it, must be taught that they can not afford to stay away. Those who are indifferent because they do not understand the true aim of these societies, and those who already know so much that they can not learn any more, must all be brought in; the latter to impart their wisdom to those less gifted. As one more means by which to secure the ultimate success of our profession, the essay advocated the education of the people.

Upon motion of *Dr. Kilbourne*—who stated that he had learned that Dr. Allport had "something new"—Dr. Allport was requested to explain his ideas before this society, and two o'clock, this P. M., was set apart for the purpose of listening to him.

*Dr. G. H. Cushing*, of Chicago, then read an essay on, "What has the Public a Right to Expect of the Dentist at the Present Day?" of which the following is an imperfect extract:

* * * A profession may be said to be a calling for which its followers are supposed to have particularly fitted themslves by long and extensive study; by means of which they have become reasonably well grounded in the great science which underlies their art, and which course of study is essential to qualify them for such calling, and is more extensive and special in its character than is required of other occupations, not called professional. The distinctive difference between practitioners of our specialty and those who follow vocations not called professional is, that the first profess to be able intelligently to treat certain diseases peculiar to a portion of the living body, and that the public are in most cases unable correctly to estimate the character of such operations and services rendered; while with almost every calling not called professional, men of ordinary intelligence can inform themselves of the quality of work which they procure to be done for them. * * * The crowning act which has marked ourprofession thus far is to be found in the acknowledgement which Harvard University has accorded to us by engrafting upon the parent stem of her medical department, ours as a recognized and honored specialty. * * *

The public then has a right to demand of the dentist at the present day, First, that he should sustain a true professional character. Such a character presupposes the dignity due to a post of such great confidence, and can never stoop to make use of means for securing public favor which, though perfectly justifiable and legitimate perhaps in matters of trade, would yet be dishonorable and unbecoming in the professional man. * * *

Second. The public have a right to expect that the dentist shall be fully educated up to the highest standard which has been attained. * * *

Further, the public have a right to expect that he apply himself to the careful study of disease, and that he shall be able more or less eventually to prevent or lessen disease, rather than repair its ravages. * * *

How, then, are we prepared to answer these demands upon us? Let each one answer for himself; and let him not think that because his patients do not say so in so many words that he is absolved from his obligations in the matter, for so long as he has the consciousness within him (and who has not?) that these are the proper claims of his patrons, he leads no truer professional life without using his best endeavors to fulfill these demands.

*Dr. Crouse*—The public has a right to expect a temperate life; a man is unfit for the proper and conscientious discharge of his duties towards his patients who indulges in excesses of any kind. * * * No one who does not attend society meetings can enter into honorable competition for excellence.

*Dr. Honsinger*—The public has a right to expect to be educated.

*Dr. Hurtt*—Some dentists hold that it is proper to advertise, in the public prints, what they would say to their patrons in private. This is objectionable; because we can not explain, as we can personally in our private offices.

*Prof. Judd* * * * , When a man publishes that he has a peculiar and superior mode of performing an operation, or that he is the best operator, and operates for less money than any body else, he does that which is beyond the pale of honor and decency. * * * He has a letter still in his possession from a Committee of the Massachusetts State Dental Society, asking for a plan of the model of the Missouri. Dental College. He complied with this request, and the plan was generally adopted in the formation of the Harvard Dental College. Hence he thinks the honor of first recognition of our specialty as a branch of medicine, is due to the St. Louis Medical College. * * * He would not detract from the just merits of Harvard Dental College, as he believed it one of the first in land, and is not sure but what it is destined to be the very first.

Dr. Cushing explained that in mentioning Harvard University he did not intend to detract from the St. Louis Medical College its just claim for priority in that direction. But Harvard is more than a mere medical school college, it is an University, and hence he used its action, to show our recognized position among the learned professions.

*Dr. A. W. Freeman*, Chicago, thought to be recognized by as old and conservative an institution, an institution so slow to sanction innovation as Harvard University, where aside from special training in medicine, jurisprudence, and divinity is also taught, is a fact well worthy of especial mention. * * * Our advertisements should mainly consist in a conscientious discharge of our duties, and although devotion to our calling, and any man with these will soon get the position that rightfully belongs to him, without recurrence to newspaper puffs.

The hour set apart for the purpose of listening to Dr. Allport having already passed, he was invited to the floor.

He illustrated upon the black-board his new theory as to the manner in which circulation, sensation, and nourishment is carried on between the dental pulp and the dentine. Said that the manner in which the functions are performed had given rise to much controversy among physiologists, histologists, and microscopists. Then proceeded to review the different theories that have been advocated, and stated that that of Tomes, showing myriads of fibrils to project from the periphery of the pulp into the dental tubuli, which fibrils are claimed by him as the means by which vitality and sensibility are maintained in the dentine, is at this time the one most generally accpted. He believed Tombs a most thorough and honest observer, but doubted the correctness of a cut contained in his system of dental surgery which represents these fibrils as protuding from the pulp to the dentine; believes the artist who drew this must have drawn largely on his imagination; the defects in which must have escaped Mr. Tomes' notice. But said, allowing the figure to be reasonably correct, its appearence can be accounted for upon other and he thought better hypothesis, than argued: Of that however he did not then propose to speak.

In giving his own ideas Dr. Allport stated that he hoped to draw out through criticism, and wished his ideas would be further investigated as he was equally ready to be convinced that he is in an error, or to have his theories confirmed by investigations of others, only that the truth be established.

His theory is that the dentine derives its nourishment and sensation from one or more points, and this point or these points being where there exists a connection or attachment between the pulp and the dentine. This connection is always where dentification first begins, and bears to the dentine a smilar relation as does the placenta to the fœtus; and that from this point vital action in the tooth is sustained. I use this term placenta for the want of a better one. Through this connection nutrition is carried from the pulp to the dentine, and sensation is transmitted from the dentine to the pulp and brain. The contents of the dentinal tubuli he believed elongated dentinal cells, endowed with nervous sensibility, anastomosing and connecting together throughout the dentine, with their origin and connection with the pulp, at the points of first dentification.

Thus he said if in a tooth with only one point of dentification or pulp attachment, this point be cut off, sensation and nourishment of the dentine would probably cease—with the exception of that which is derived from the alveoli dental periosteum—though the pulp itself may still be living with teeth with more than one part of dentification or attachment, all the attachments must be severed before sensation is destroyed in the entire dentine. Thus by severing the attachments at one point only of the molar, sensation will be destroyed in that portion of the tooth only. He has exhibted specimens showing this attachment at the points of dentification to Drs. Judd, Willson, Morrison, Cushing, Dean, and others.

*Dr. Honsinger*—How can amputation of part of the nerve then be performed without endangering the life of the tooth?

*Dr. Allport*—It would endanger the life of the tooth but not make it certain.

If, however, a piece be cut out of the *side* of the pulp and the attachment with the dentine not interfered with, the pulp might heal, and the vitality of the dentine still be preserved A wounded pulp under favorable circumstances will heal nearly as readily as a wound in any other part of the body.

*Dr. Kennicott*—Suppose the tooth becomes worn down by attrition, and a secondary dentine deposited at this placental point, and in this attrition the fibres conveying sensation to this point become also obliterated, how would the life of the tooth be effected?

*Dr. Allport*—Should think that the point of connection would recede the same as it does in the original dentification, unless the attrition was so rapid that nature could not adapt itself to the new condition. See nothing in this that would necessarily refute this theory. Nature is not only an industrious, but an ingenious worker.

*Dr. Black*—How is nutrition carried on throughout the whole dentinal substance?

*Dr. Allport*—If this theory is correct, from the point of cell attachment with the pulp, through their anastomosing connection throughout the dentine.

*Prof. Judd*—This idea is certainly a new and a very ingenious one. The existence of this connection has never had this importance attached to it by any writer on histology before; and he does not think that it must necessarily follow that all nutrient matter must first pass this point because of this connection betwen pulp and dentine. The fluid oscillation theory has been long since exploded; he has been content to believe that there are filaments passing through the tubuli and anastomosing branches from the whole surface of the pulp. Whilst he was not prepared to condemn this new theory, should be very slow to believe it until it has been more thoroughly investigated. It will require very strong proof to show that so careful and honest an observer as Tomes was mistaken.

There is no need in reasoning upon what is demonstrable. Hopes histologists will investigate this matter more thoroughly as it is capable of being demonstrated. He hoped that Dr. Allport would give to the profession an elaborate paper, explaining his theory.

Essay on Dental Fees was then read by Dr. Hurtt, of Peoria, but owing to the lateness of the hour was not discussed.

The Society then proceeded to the election of officers and the transaction of other business, after which it adjourned to meet at Rock Island, in May, '73.

The Illinois State Dental Society publishes its transactions annually in pamphlet form, as early as practicable after the respective meetings; full copies of which, and of the session especially, may be obtained of the Secretary, Dr. Chas. R. E. Rach. Price, 50cts. per copy. ED. REG.

